# Alcohol-Related Frequent Attenders to Emergency Departments: A Scoping Review with Implications for Singapore

**DOI:** 10.3390/jcm15134892

**Published:** 2026-06-23

**Authors:** Juntian Wu, Marcus Eng Hock Ong, Desmond Renhao Mao, Mikael Hartman, Xueling Sim, Benjamin Sieu-Hon Leong, Rachel Siying Lee, Fahad Javaid Siddiqui

**Affiliations:** 1 Pre-Hospital and Emergency Research Centre (PERC), Duke-NUS Medical School, 8 College Road, Singapore 169857, Singapore; fahad.siddiqui@duke-nus.edu.sg; 2 Emergency Department, Singapore General Hospital, Outram Road, Singapore 169608, Singapore; marcus.ong@duke-nus.edu.sg; 3 Acute And Emergency Care, Khoo Teck Puat Hospital, 90 Yishun Central, Singapore 768828, Singapore; mao.desmond.r@nhghealth.com.sg; 4 Division of General Surgery (Breast Surgery), Department of Surgery, National University Hospital, 5 Lower Kent Ridge Road, Singapore 119074, Singapore; ephbamh@nus.edu.sg; 5 Saw Swee Hock School of Public Health, National University of Singapore, 21 Lower Kent Ridge Road, Singapore 119077, Singapore; ephsx@nus.edu.sg (X.S.); e0555804@u.nus.edu (R.S.L.); 6 Emergency Medicine Department, National University Hospital, 5 Lower Kent Ridge Road, Singapore 119074, Singapore; benjamin_sh_leong@nuhs.edu.sg

**Keywords:** alcohol-related frequent attenders, emergency department, scoping review, healthcare utilisation, substance use disorder, case management, assertive community treatment, intervention effectiveness

## Abstract

**Background/Objectives**: Alcohol-related frequent attenders (ARFAs) constitute a small but resource-intensive emergency department (ED) population. **Methods**: Following PRISMA-ScR guidelines, we searched MEDLINE, PsycINFO, CINAHL Complete, and EMBASE from inception to May 2025 for empirical studies examining ED frequent attendance with alcohol involvement. Definitions had high heterogeneity; therefore, narrative synthesis was conducted. **Results**: A total of 73 studies were included, most retrospective (57.5%), encompassing sample sizes from 14 to over 4.1 million participants: 59 frequent attender (FA) studies with alcohol subgroup analyses and 14 pure ARFA studies. Research was concentrated in North America and Europe (56/73, 76.7%), with limited Asia-Pacific representation (21.9%). Seven distinct definition threshold categories were identified (≥2 to ≥20 visits annually); 31.5% utilised different definitions. Qualitative studies (n = 6) identified push factors (dependence, mental health crises, housing instability, fragmented services) and pull factors (24/7 access, crisis care model, immediate service) driving frequent attendance. Eight studies evaluated interventions; all employed non-randomised designs examining case management, integrated pathways, and community-based treatments. **Conclusions**: Critical gaps include the absence of standardised definitions for comparison across studies, a concentration of research in Western settings limiting global applicability, and insufficient rigorous intervention evidence. Priorities include developing empirically validated definitions, expanding non-Western research, and conducting randomised controlled trials with adequate follow-up.

## 1. Introduction

Emergency departments (EDs) worldwide face mounting pressures from patients who attend repeatedly throughout the year. These frequent attenders (FAs), although representing only 1–5% of all patients, account for up to 20% of total emergency visits and contribute disproportionately to healthcare expenditure [1,2]. Within this population, there is a clinically distinct subgroup—alcohol-related frequent attenders (ARFAs)—whose recurrent presentations stem primarily from alcohol use disorders (AUDs) and their complications. Unlike general FAs whose multiple visits may result from various chronic conditions, ARFAs present with a characteristic pattern of acute intoxication, withdrawal syndromes, trauma, and exacerbations of alcohol-related chronic diseases [3,4].

The global burden of alcohol-related ED presentations has expanded substantially over the past two decades. In the United States, research data document a 61.6% increase in alcohol-related ED visits between 2006 and 2014 [5]; in similar contexts, England recorded approximately 280,000 alcohol-specific admissions in 2019–20 [6]. Singapore also experienced a tremendous increase in alcohol-related emergency presentations between 2007 and 2016; there was a rise of 98% from 2236 to 4433 cases, with a 140% escalation in associated costs [7]. These trends reflect broader patterns across diverse cultural and healthcare settings, which may suggest that ARFA management could represent a universal challenge and not just a localised phenomenon [8,9].

ARFAs’ acute care utilisation tends to be in cyclical patterns that might differ fundamentally from typical FA populations. ARFAs’ ED presentations involve immediate medical crises: acute intoxication, withdrawal syndromes, traumatic injuries, or decompensation of chronic conditions [3,4]. As a result, management complexity should extend beyond normal medical issues to encompass intersecting social vulnerabilities, for example, mental health comorbidities, housing instability, unemployment, and social isolation [4,7]. On the other hand, economic data show both the substantial expenditure and poor outcomes for this population. The Ontario cohort study demonstrates a 5.4% overall one-year mortality among patients with frequent alcohol-related ED visits, and the number rises to 8.8% among those with five or more annual visits [10]. These figures highlight the medical complexity and mortality risk that distinguish ARFAs from general FAs.

Despite a growing recognition of ARFAs as a distinct clinical population different from FAs, ARFA research remains fragmented. Individual studies have examined various aspects of frequent ED attendance, but comprehensive reviews that specifically focus on ARFAs have been lacking. Existing systematic reviews are mostly on general FAs and treat alcohol-related cases as subgroups without examining their unique characteristics [11,12]. Understanding these potential differences is fundamental to designing targeted interventions and forms a core rationale for the present review. Preliminary observations suggest considerable variation in how ARFAs are defined and studied across the literature, but no systematic synthesis has examined the scope of this heterogeneity or its implications for clinical practice and policy.

The geographic distribution of published FA studies with an ARFA subgroup and pure ARFA research suggests Western healthcare system predominance. It remains unclear whether the available evidence adequately represents the range of cultural, economic, and healthcare system contexts in which ARFAs present. Given that alcohol-related harm is shaped by cultural norms, religious practices, and healthcare organisation, understanding the breadth and distribution of existing research is essential before drawing conclusions about the generalisability of current findings.

It is unknown if the patterns documented in North American and European contexts apply to different cultural, economic, and healthcare systems in Asia. Meanwhile, the intervention evidence base appears similarly limited. While some intervention studies targeting ARFAs or related populations exist, their diversity in focus, methods, and settings makes it difficult to gauge the full scope of available evidence. It is unclear whether the more visible published trials represent the totality of interventions attempted or whether additional unpublished or less accessible evaluations might alter our understanding of what works for this population.

It is essential to understand what interventions could effectively address ARFA needs, or whether there are sustainable interventions over time, and their cost-effectiveness compared to usual care. Without a systematic understanding of ARFA characteristics and effective interventions, healthcare systems worldwide will continue to provide fragmented, costly care that fails to address the underlying causes of repeated ED attendance. It is thus also critical to address this research gap considering the rising global alcohol consumption and growing recognition that conventional ED practices are inadequate in addressing the complex psychosocial needs of ARFAs, as ARFAs receive largely crisis-oriented reactive care instead of holistic, prevention-focused interventions [9].

This scoping review aims to answer the knowledge gap through two primary aims: firstly, this review aims to establish the volume and variety of research dedicated to ARFAs; secondly, this review aims to examine study characteristics that include methodological approaches, geographical distributions, and thematic focuses. The findings of both of these aspects will identify worthwhile research focus areas on this topic.

## 2. Materials and Methods

We followed Preferred Reporting Items for Systematic reviews and Meta-Analyses extension for Scoping Reviews (PRISMA-ScR) [13,14]. The protocol was prospectively registered on Open Science Framework (OSF: https://osf.io/y8sg2, accessed on 12 October 2025).

We searched four electronic databases: MEDical Literature Analysis and Retrieval System ON-LINE (MEDLINE, via PubMed), Psychological Information (PsycINFO), Cumulative Index to Nursing & Allied Health Literature (CINAHL) Complete, and Excerpta Medica dataBASE (EMBASE) from inception to 30 May 2025. These databases provide coverage of medical, psychological, nursing, and health policy literature relevant to emergency medicine and addiction research. Scopus and Web of Science were not searched; these multidisciplinary citation indexes overlap largely with MEDLINE and EMBASE for biomedical content, and the four selected databases were prioritised for their subject-specific depth across emergency medicine, psychology, nursing, and health services research (HSR). Our search strategy combined both medical subject headings (MeSH) and free-text keywords.

Inclusion criteria included (a) pure ARFA studies where the entire cohort comprised alcohol-related ED patients and (b) FA studies reporting alcohol-specific subgroups or outcomes. All quantitative, qualitative, and mixed methods studies published in English were eligible. Exclusion criteria were non-English publications; conference abstracts, editorials, commentaries, and narrative reviews without systematic methodology; studies not examining alcohol involvement among frequent attenders; and case reports with fewer than five patients. English-only restriction was applied due to feasibility constraints. Non-English publications were excluded due to the lack of translation capability within the research team.

Pure ARFA studies were defined as investigations exclusively examining ARFAs. FA studies with alcohol subgroups included broader research reporting alcohol-specific outcomes or subgroup analyses. Alcohol case ascertainment methods, as reported across the included studies, encompassed ICD-9 and ICD-10 diagnostic codes, SNOMED clinical terminology, clinical documentation in medical records, blood alcohol concentration or toxicology screening results, and clinician or provider assessments.

For consistency, the following terminology is used throughout this paper: “pure ARFA studies” denotes studies in which the entire cohort comprised ARFAs, and ‘FA + ARFA’ denotes FA studies that report alcohol-specific subgroups or outcomes.

Study selection employed systematic three-stage screening using Covidence (Veritas Health Innovation, Melbourne, VIC, Australia), a web-based systematic review management platform. Two reviewers screened titles, abstracts, and full texts independently. Disagreements were resolved through structured discussion with a third reviewer [13].

Data extraction used Covidence charting functionality with customised forms developed iteratively through team consensus. Domains included: study characteristics (country, setting, design, sample size, follow-up duration), definitional approaches (frequent attendance thresholds, alcohol case ascertainment methods), participant demographics and clinical characteristics, social determinants, outcomes (utilisation patterns, admissions, mortality, costs), and intervention effects where applicable. Two reviewers extracted data independently, with discrepancies resolved through discussion and senior reviewer consultation when necessary.

Narrative synthesis mapped concepts, identified patterns, and characterised evidence gaps. Considerable definitional and methodological heterogeneity precluded meta-analysis. The approach involved tabulation of study characteristics and outcomes, thematic analysis of patterns, and structured gap analysis. Given heterogeneity in definitions, time windows, and ascertainment methods, summary statistics are presented as median with range where applicable rather than pooled estimates. Studies with small samples were retained deliberately to be consistent with scoping review methodology: in order to map the full breadth of evidence in an emerging field, their findings were interpreted as preliminary or feasibility evidence, and therefore, they should not be weighted equally with large administrative cohorts.

Qualitative data were synthesised thematically. We adopted the push–pull framework used across the primary qualitative studies, where push factors represent external pressures driving individuals toward ED use and pull factors represent ED characteristics that encourage repeated attendance.

This study is supported by the Khoo Teck Puat Health Fund through the Science-Translational & Applied Research (STAR) grant, STAR22102.

## 3. Results

### 3.1. Study Selection

**Figure 1:**
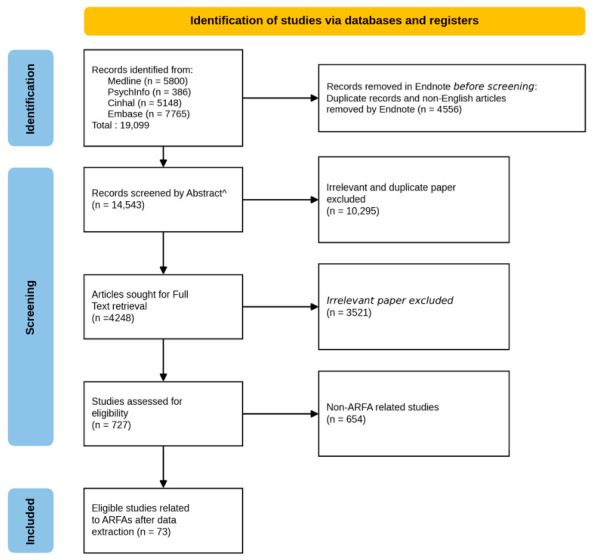
PRISMA flowchart of included papers.

The systematic search identified 19,099 records across four databases: MEDLINE (n = 5800), PsycINFO (n = 386), CINAHL Complete (n = 5148), and EMBASE (n = 7765). Following compilation and automated deduplication, 4556 duplicate and non-English records were removed, leaving 14,543 unique citations for screening. Title and abstract screening excluded 10,295 records. The remaining 4248 articles underwent full-text retrieval, with 4175 excluded for various reasons, including absence of alcohol subgroup analysis, inappropriate population, insufficient detail, or other methodological concerns. Seventy-three studies met all inclusion criteria for data extraction and synthesis (Figure 1; Table S1). The included studies comprised approximately 11.4 million participants in total, although sample sizes varied enormously from 14 to over 4.1 million, which reflected the mix of targeted clinical cohorts and large population-level administrative database analyses. However, this cumulative figure should be interpreted with caution. It is dominated by twelve large administrative datasets and may overlap between studies that draw on the same national or regional databases. The cumulative sample size therefore reflects the scale of data examined and should not be treated as a signal of evidentiary strength.

Twelve studies utilised national or regional administrative datasets that exceeded 100,000 records each; the remaining 61 studies had sample sizes ranging from 14 to 82,046 (median: 325).

### 3.2. Study Characteristics

**Figure 2:**
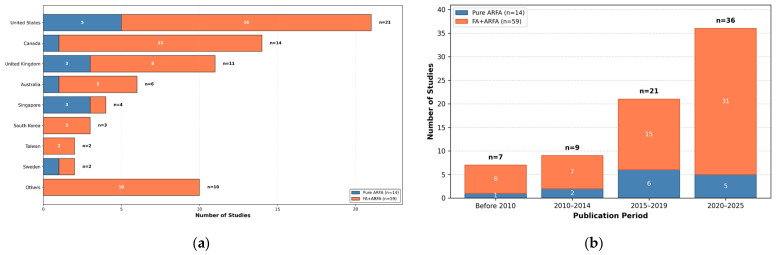
Study distribution by geography and publication period (n = 73). (**a**) Geographic distribution of included studies showing concentration in Western settings (North America and Europe); (**b**) temporal trends showing increasing publication activity, with nearly half of all studies (49.3%) published between 2020 and 2025. Blue bars represent pure ARFA studies (n = 14); orange bars represent FA with alcohol subgroups (FA + ARFA, n = 59).

Out of 73 included studies, there were 14 pure ARFA studies (19.2%) and 59 FA + ARFA (80.8%, Table 1 and Figure 2). Most included studies originated from Western countries. North America contributed 47.9% (35/73), including 21 studies from the United States (US) and 14 from Canada. Europe accounted for 28.8% (21/73), with the United Kingdom (UK) contributing 11 studies. Asia-Pacific regions represented 21.9% (16/73), including Singapore (n = 4), South Korea (n = 3), Australia (n = 6), Taiwan (n = 2), and Hong Kong (n = 1). One study (1.4%) originated from other regions or involved multiple countries. Among pure ARFA studies specifically, six originated from North America (42.9%), four originated from Europe (28.6%), and four originated from Asia-Pacific (28.6%). This geographic concentration raises concerns about external validity when applied to non-Western healthcare contexts. This geographic concentration partially mirrors global per-capita alcohol consumption patterns—higher consumption documented in the WHO European and Americas regions. However, rising alcohol consumption in the WHO Western Pacific and South-East Asia regions, including documented increases in countries such as Singapore, China, and India, suggests that the current Western-centric evidence base may inadequately represent emerging alcohol-related public health challenges in these populations.

Most of the studies were retrospective cohort designs (57.5%), followed by cross-sectional studies (15.1%), non-randomised controlled trials (11.0%), and qualitative investigations (8.2%). The remaining employed mixed methods (4.1%), case-control designs (2.7%), and prospective cohorts (1.4%). Among pure ARFA studies, six used retrospective cohorts (42.9%), and the other five were qualitative designs (35.7%), two non-randomised controlled trials (14.3%) and one prospective cohort (7.1%). The preponderance of retrospective designs reflects reliance on administrative data but limits causal inference to show intervention effectiveness.

Publication activity had a steady increase in recent years (Figure 2). Nearly half of all studies (49.3%) were published between 2020 and 2025. Earlier periods contributed fewer studies: 21 (28.8%) from 2015 to 2019, nine (12.3%) from 2010 to 2014, and only seven (9.6%) before 2010. Pure ARFA studies showed similar temporal distribution. This temporal pattern suggests growing recognition of ARFAs as a distinct population for dedicated investigation.

### 3.3. Threshold Definitions

Definitional heterogeneity represented one of the most important findings. Seven distinct threshold categories were identified, ranging from two or more to 20 or more annual visits (Table 2).

The most common explicit thresholds were ≥4 visits/year (21.9%) and ≥5 visits/year (16.4%). Higher thresholds of 10–19 visits/year appeared in six studies (8.2%). Lower thresholds of 6–9 visits/year were used by three studies (4.1%). Notably, 23 studies (31.5%) used alternative approaches, which included percentile-based definitions, top N patients, range-based classifications, algorithm-based methods, or did not specify any threshold.

On the other hand, we also noticed 32 studies (43.8%) provided no explicit rationale for their chosen threshold. Among studies providing justification, the most common approach was citing prior literature as precedent, without empirical validation of threshold appropriateness for the specific study population or healthcare context.

Pure ARFA studies tended toward higher thresholds than general FA studies (Table 2). Among pure ARFA studies, 28.6% (4/14) used thresholds of ≥10 visits annually compared with only 3.4% (2/59) of FA + ARFA. Among the 16 studies using ≥4 visits/year, only one was a pure ARFA study, and 15 were FA + ARFA studies. Similarly, for ≥5 visits/year (n = 12), only two were pure ARFA studies, while 10 were FA + ARFA studies.

Most studies (61/73, 83.6%) defined frequent attendance on an annual basis. The remaining 12 used alternative time frames, including cumulative visits across multi-year study periods, 72 h repeat visit windows, percentile-based or top N rankings without fixed time periods, and combined criteria that incorporated both annual and sub-annual thresholds (e.g., ≥10 visits/year or ≥5 visits/3 months). Where combined criteria were used, classification in this review was based on the primary annual threshold.

Across the 73 included studies, alcohol involvement was identified through ICD-10 diagnostic codes (n = 15), ICD-9 codes (n = 11), clinical documentation and electronic health records (n = 14), clinician or provider assessments (n = 4), and national classification systems such as KCD-5 and SNOMED-CT (n = 3). Four studies reported multiple ascertainment methods; therefore, categories are not mutually exclusive. A substantial proportion of studies (n = 26, 35.6%) did not explicitly state their method of ascertaining alcohol involvement, which limits reproducibility and comparability across the evidence base.

### 3.4. Qualitative Insights: Understanding Repeated Attendance

Six qualitative investigations employing semi-structured interviews examined reasons behind frequent attendances (Table 3) [15]. These studies were conducted across four countries (UK, US, Singapore, Canada), with sample sizes that ranged from 20 to 30 participants. Thematic analysis and framework analysis were mainly used to explore patient experiences.

The qualitative review showed that repeated ED attendance was driven by a combination of push and pull factors that interacted with each other over time.

Push factors were external pressures that pushed individuals toward the ED. These factors included alcohol dependence: withdrawal effect, acute intoxication, and traumatic injuries, which often caused urgent medical problems that community services were unable to manage effectively. Many patients also had co-existing mental health conditions, which made it difficult for them to engage with standard treatment pathways. Housing instability frequently triggered acute crises that required emergency care, and social isolation and histories of trauma further compounded vulnerability. On the other hand, fragmented health and social care systems, poor coordination between providers, and past experiences of feeling judged by healthcare staff created barriers to accessing non-emergency services [16,17,18,19].

**Table 3:** Characteristics and findings of qualitative studies examining ARFAs (n = 6).

Author (Year)	Sample Size	Study Objective	Key Findings/Themes
Parkman (2017) [18]	30	Explore the use and experience of specialist addiction services	High ED usage with low addiction service engagement; 11/30 identified alcohol-specific treatment as desired support; participants wanted help with mental health, social contact, work, housing, or gym access
Parkman (2017) [19]	30	Understand reasons for repeated ED attendance to reduce unnecessary demands on hospital resources	Long-standing health problems; positive health beliefs about EDs despite negative experiences; limited community resources (poor social support, inaccessible primary care, difficulties with specialist addiction services); physical injury and pain as main reasons for attendance
Neale (2017) [16]	30	Explore socio-demographic characteristics and stereotyping of ARFAs	Years of heavy drinking; high mental and physical illness; unemployment; state benefits dependence; housing problems; social isolation; varied attendance frequency and diverse drinking patterns
McCormack (2015) [17]	20	Describe evolution and psychosocial context of alcoholism from homeless frequent ED users’ perspective	Four major themes identified: alcoholism, homelessness, healthcare, and future; multifactorial process for alcoholism evolution; bidirectional reinforcing relationship between alcoholism and homelessness; progressive hopelessness eroded self-efficacy
Goh (2022) [20]	20	Explore reasons for repeated ED/EMS utilisation and perpetuating and protective factors for drinking	Perceived need due to symptoms and bystander calls; ED/EMS preferred for perceived higher quality and speed of care; persistent drinking attributed to social and environmental factors and as coping mechanism; rehabilitation programs and meaningful activities reduced drinking
Samosh et al. (2024) [15]	21 (15 clients; 6 case managers)	Investigate perceived outcomes of community mental health service combining system navigation and intensive case management	Perceived reduction in ED use, mental illness symptom severity, and improved quality of life; mixed addiction outcomes; positive working relationships between clients and case managers, and focused skill development identified as key mechanisms

ARFA = alcohol-related frequent attenders; ED = emergency department; EMS = emergency medical services. Two Parkman (2017) studies represent separate investigations with distinct research questions on different aspects of ARFA experiences.

Pull factors were features of the ED that encouraged repeated use. Participants described the ED as readily accessible: 24/7 service without appointments. The crisis-oriented model of care aligned well with their immediate needs, and the ED environment was sometimes perceived as less judgmental than other healthcare settings. Repeated attendance was reinforced by easy access to ambulance transport, rapid treatment without delays from insurance checks or referral processes, and the ED’s role as a dependable safety net when other services were unavailable. Across studies, participants consistently emphasised that the ED was one of the few places where they could obtain prompt care without bureaucratic obstacles [19,20].

A key question across qualitative studies was why participants did not access non-emergency services despite their recurrent health needs. Several barriers were identified: chronic unemployment, unstable housing, and competing survival priorities often took precedence over engagement with addiction treatment. Many individuals described alcohol use not as recreational but as a coping strategy in response to homelessness, past trauma, and social isolation. Treatment trajectories were consequently hindered by frequent disengagement, and housing instability and mental health crises repeatedly emerged as obstacles to sustained participation in addiction care.

Abstinence-only approaches appeared insufficient for reducing frequent ED attendance in this population because alcohol served multiple survival functions. Across qualitative studies, participants consistently described drinking as a coping mechanism for difficult living conditions, trauma, and social isolation—functioning as self-medication, a social connector, or a sleep aid. Asking patients to cease drinking without offering alternatives to address these underlying needs risks worsening their situation. This pattern appeared consistently across different healthcare systems and geographic settings and suggested a common mechanism and not just a culturally specific phenomenon.

### 3.5. Intervention Evidence

Eight studies (11.0% of all included studies) evaluated interventions targeting ARFAs or FAs with alcohol subgroups (Table 4). The intervention evidence base was dominated by non-randomised controlled trial designs that examined pre–post treatment effects. All eight intervention studies employed before–after comparisons without randomisation, limiting confidence in causal inference.

Case management approaches were evaluated across multiple settings (n = 4), with varied results. Chiang et al. assessed dynamic internet-mediated, team-based case management in Taiwan (n = 14) and reported a 58.3% reduction in mean ED visits (10.5 to 4.36 visits/patient/month, *p* = 0.004) [21]. Hedayioglu evaluated pilot case management services in the United Kingdom (n = 24) and demonstrated the value of the approach, though specific quantitative results were not fully reported [22]. Sathyanarayanan examined focused case management in the United States (n = 29), finding significant reductions in both ED visits (0.52 to 0.31 per patient/month, *p* < 0.001) and inpatient visits (0.10 to 0.03 per patient/month, *p* < 0.01) [25]. Elston evaluated telephone-based case management in the United Kingdom (n = 808), reporting moderate effectiveness, with incidence rate ratios of 0.856 for emergency department attendance and 0.871 for admissions at 12 months, though overall impact on total emergency department activity was small [26].

Integrated pathway approaches linking EDs with addiction services demonstrated significant outcomes. Lintzeris et al. evaluated the IMPACT (Integrated Management Pathways for Alcohol and Drug Clients into Treatment) programme in Australia (n = 46, 34 intervention, 12 comparison) and documented significant reductions in preventable ED presentations (*p* < 0.05), non-preventable ED presentations (*p* < 0.01), hospital admissions (*p* < 0.01), associated costs, and primary substance use days (*p* < 0.01). Scheiner described RAID (Rapid Access, Interface & Discharge) team interventions in the UK (n = 101) and reported highly significant mean reduction in ED attendances (*p* < 0.0001), though the effects were smaller for patients with alcohol-related presentations compared to other FAs [24].

Assertive Community Treatment (ACT) models adapted for ARFAs showed promising outcomes in Asian contexts. Mak et al. piloted ACT intervention in Singapore (n = 14), reported a 45.3% reduction in ED visits post-intervention (*p* = 0.025) and showed feasibility of the ACT model for ARFA populations [28]. Wu et al. reported a subsequent follow-up ongoing protocol for evaluating ACT effectiveness at a larger scale and cost-effectiveness in Singapore public hospitals (n = 93 ARFAs recruited from three hospitals); the outcomes are pending trial completion [27].

### 3.6. Conceptual Framework

**Figure 3:**
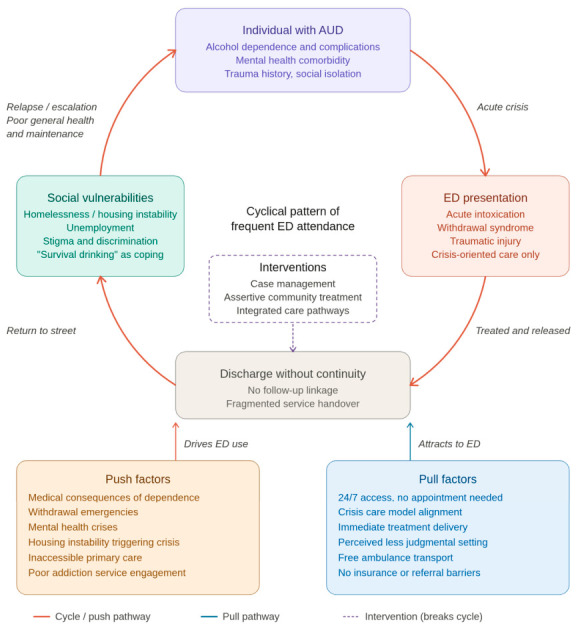
Conceptual framework of cyclical ARFA attendance at ED from 73 included studies.

Based on the findings of the included 73 studies, we developed a conceptual framework to illustrate the cyclical pattern of alcohol-related frequent ED attendance (Figure 3). The framework depicts how alcohol patients enter the ED during acute crises—intoxication, withdrawal, or traumatic injury—and how they receive crisis-oriented treatment and are discharged without continuity of care or follow-up linkage. Upon return to the community, pre-existing social vulnerabilities that include homelessness, unemployment, stigma, and fragmented services remain unaddressed, perpetuating a pattern of “survival drinking” as a coping mechanism and contributing to poor general health maintenance that drives re-presentation. Push factors that we identified through qualitative synthesis would serve as external pressures that compel individuals toward the ED. On the other hand, pull factors reflect features of the ED itself: round-the-clock access, immediate care without bureaucratic barriers, and a perceived non-judgmental environment. Those pull factors reinforce repeated use. Intervention approaches, such as case management, assertive community treatment, and integrated care pathways, tend to target the transition between discharge and community return and aim to disrupt the cycle by establishing continuity of care and addressing underlying psychosocial needs. This framework provides a foundation for designing and evaluating future ARFA-specific interventions and may guide healthcare systems in identifying the most effective points for breaking the cycle of repeated attendance.

## 4. Discussion

Our scoping review is one of the first that systematically mapped global evidence on ARFAs through synthesis of 73 studies published from 1981 to 2025. Fourteen pure ARFA studies and 59 FA + ARFA studies were identified. Our results indicate ARFAs are predominantly examined as subgroups within broader FA research, while this warrants dedicated investigations. Our findings represent an important observation about the current state of the field: the relative paucity of pure ARFA studies suggests this clinically important population has received insufficient focused attention despite their substantial healthcare utilisation and poor outcomes.

The characteristics of the included studies reveal several findings with direct implications for policy and service design. Most research was conducted in Western settings (76.7% of studies originating from North America and Europe). More than half of the studies used retrospective designs (57.5%). This concentration in high-income Western healthcare systems may affect the external validity of the existing evidence base. Alcohol-related harm, family and community support structures, healthcare organisation, and help-seeking behaviours are strongly shaped by cultural and system-level contexts. As a result, findings derived largely from Western populations may only be partially applicable to Asian and other regions. Locally generated evidence is therefore critical for evaluating the adequacy and effectiveness of current policies on ARFAs.

On the other hand, according to WHO’s data, Africa, China, and India collectively account for a significant share of global alcohol consumption and alcohol-attributable harm; however, these nations contributed little to our evidence base [29,30]. The shortage of their research contribution could be due to research constraints or publication barriers and not because there are no ARFAs there. As a result, we face another blind spot for global health policy.

Definitional heterogeneity emerged as the most fundamental barrier to both scientific progress and clinical implementation. While this partly reflects higher per-capita alcohol consumption in these regions and more established research infrastructure, it means the existing evidence base may not fully apply to healthcare systems in Asia, Africa, and the Middle East, where alcohol consumption patterns, cultural contexts, family support structures, and healthcare organisation differ substantially.

Such wide variation undermines meaningful comparison across studies and creates practical difficulties for health systems to implement operational interventions. When a programme defines frequent attendance at two visits per year and another at more than ten, service planning, benchmarking, and evaluation become inherently unstable. This creates uncertainty for designing interventions, as researchers may have issues figuring out who should be targeted. Our review also suggests that alcohol-related frequent attendance may not follow the same definitional logic as general frequent attendance. Threshold choices differed between pure ARFA studies and general FA studies. For example, 28.6% of pure ARFA studies used thresholds of ≥10 annual visits compared with only 3.4% of FA studies that included alcohol as a subgroup. From a policy perspective, this finding supports the development of ARFA-specific identification frameworks; blindly adapting FA thresholds might misclassify risk and dilute intervention impact.

There were not many interventional studies, but consistent themes and patterns were observed. Relationship/rapport-based, community-focused approaches appeared more promising than one-off, single-encounter interventions. Case management showed beneficial effects across multiple settings, although implementation models and outcomes varied widely. The strongest signals came from intensive approaches aligned with Assertive Community Treatment (ACT) principles: proactive outreach in the community, smaller caseloads, sustained relationship building, and shifting care delivery away from clinic-centred models. Several trials reported quantitative reductions in ED attendance. Chiang et al. documented a 58.3% reduction in mean ED visits (*p* = 0.004), Sathyanarayanan et al. reported significant decreases in both ED visits (*p* < 0.001) and inpatient visits (*p* < 0.01), Lintzeris et al. demonstrated reductions in preventable and non-preventable ED presentations alongside significant cost savings (*p* < 0.01), and Mak et al. reported a 45.3% reduction in the Singapore ACT pilot (*p* = 0.025) [21,23,25,28]. The Singapore finding is notable as it demonstrates feasibility in an Asian healthcare context, but the small sample (n = 14) and pre–post design limit causal inference, as with all included intervention studies. Only Lintzeris et al. reported associated cost reductions; no study conducted a formal cost-effectiveness analysis, representing a critical evidence gap [23].

Our findings could directly inform national strategies that ED-driven identification combined with community-based, longitudinal engagement may be a viable pathway for managing high-risk ARFAs at scale.

However, all intervention studies used non-randomised designs, which limits causal inference. Reductions in utilisation may partly reflect regression to the mean, as patients are often referred into programmes during peaks of their attendance and it is possible that they may naturally revert to lower levels over time. Selection bias is also likely. Individuals who engage with programmes may differ from those who decline in ways that predict better outcomes. The absence of control groups also makes it difficult to separate intervention effects from broader influences, such as policy changes, service redesign, or secular trends in healthcare use. In almost all interventions, follow-up was usually short (typically ≤12 months); hence, the durability of reported improvements remains uncertain.

A particularly important qualitative insight was the concept of “survival drinking.” Abstinence-based approaches may be unrealistic and potentially harmful if basic survival needs remain unaddressed when alcohol is used to cope with trauma, homelessness, and chronic isolation instead of recreation [17,31]. For many patients, harm-reduction strategies that accept continued alcohol use while aiming to reduce associated harms may be more appropriate than abstinence-focused models [32,33].

The existing literature is notably silent on financial determinants of ARFA ED attendance patterns. No included study systematically examined the role of health insurance coverage, out-of-pocket payment requirements, or immigration status in shaping ED utilisation among ARFAs. The existing literature also lacks information on ARFAs from higher socioeconomic groups. Patients with better financial resources may have access to private healthcare, which make them largely invisible in studies that rely on public hospitals’ administrative data. This affluent group of ARFAs remains unexplored, and it may affect the population-level burden estimation. These omissions limit our understanding of how system-level financial structures influence who becomes an ARFA and who accesses alternative care pathways.

Locally generated ARFA evidence matters for Singapore. Nationally, alcohol-related ED attendances rose by 98% between 2007 and 2016, with a 140% escalation in associated costs [7]. The four Singapore studies included in this review together outline the local ARFA profile. Frequent EMS users with alcohol-related diagnoses were predominantly younger males with prominent social vulnerabilities: smoking, substance use, and unpaid medical debt. Local ARFAs were frequently conveyed from public areas and were less likely to get admitted. Qualitative work confirmed that local ARFAs preferred ED and EMS care for its perceived speed and quality and attributed persistent drinking to social and environmental circumstances over recreation. These patterns mirror the push–pull dynamics identified internationally. They suggest that the cyclical attendance framework developed in this review applies in the Singapore context despite differences in healthcare financing and a lower population prevalence of homelessness.

Singapore also contributed two of the eight intervention studies: the ACT pilot, which demonstrated a 45.3% reduction in ED visits and the feasibility of community-based assertive treatment for ARFAs, and an ongoing multi-hospital trial that evaluates effectiveness and cost-effectiveness at scale. There are three policy implications. Firstly, ARFA identification can leverage Singapore’s centralised administrative and EMS data systems, but it requires an agreed local threshold definition; our finding that pure ARFA studies adopt higher thresholds than general FA studies cautions against simply importing general FA cut-offs. Secondly, the consistency of “survival drinking” across settings suggests that abstinence-only pathways are unlikely to reduce attendance in this population, and harm-reduction, community-based models are more promising. Lastly, ARFA management involves healthcare, social services, and public health agencies; thus, economic evaluation of ongoing and future interventions should not be based on hospital-only approaches but should adopt a whole-of-government perspective.

Our review has several strengths. Comprehensive searches across multiple databases ensured broad coverage of emergency medicine, addiction, and HSR. Importantly, we separated pure ARFA studies from FA research, established a distinct evidence base for ARFAs, and revealed important differences in threshold selection and study characteristics. Integrating both quantitative and qualitative evidence allowed a more complete understanding of utilisation patterns, patient experiences, and intervention mechanisms. Together, these strengths support the relevance of this review’s findings for informing locally tailored identification strategies, community-based intervention design, and future evaluation priorities within Singapore’s integrated healthcare system.

However, we also faced limitations in our review. Firstly, restricting inclusion to English-language publications may have excluded relevant studies from non-English-speaking countries. This restriction is particularly consequential for the present review: excluded non-English studies are most likely to originate from the very regions under-represented in our findings, and the observed concentration of evidence in Western settings may therefore be partly reinforced by the language criteria. Secondly, grey literature was not searched. Unpublished service evaluations and interventions may therefore have been missed, and this may contribute to the limited intervention evidence base observed in this review. Future updates should consider grey literature sources to capture such evaluations.

Thirdly, the final search was conducted on 30 May 2025; given the accelerating publication activity in this field, relevant studies published after this date may not be captured, and an updated review will be needed as the evidence base matures.

Fourthly, publication bias likely affects the intervention literature, as studies that showed positive results are more readily published than those reporting null findings. Lastly, four included studies were partially authored by members of the current research team. This potential conflict of interest is openly disclosed; these studies were identified through the systematic search and were screened and extracted using identical procedures as all other included studies.

Several immediate research priorities emerge from this review. Development of standardised yet flexible ARFA definitions should have the highest priority. Such definitions require validation across different healthcare contexts and patient populations to establish whether universal thresholds are appropriate or context-specific definitions are necessary. This work should address the current situation where 31.5% of studies used alternative approaches that defy categorisation and where pure ARFA versus FA + ARFA studies show substantially different threshold patterns. Future definitional work should also consider whether visit frequency alone is sufficient to identify ARFAs. Our findings suggest that identification may be strengthened by multidimensional criteria that anchor a frequency threshold to standardised alcohol-attributable diagnosis ascertainment and that incorporate clinical markers and psychosocial complexity, such as admission risk, mortality risk, or healthcare costs. Composite definitions of this kind would better reflect the clinical reality of this population than visit counts alone, although they will require empirical validation across healthcare settings. Standardised ascertainment of alcohol involvement is as fundamental as standardised visit-frequency thresholds. In this review, not all included studies explicitly report how alcohol involvement was identified, which risks misclassification and systematic underestimation of the ARFA burden. Future studies should report ascertainment methods explicitly and consensus on preferred approaches. For example, diagnostic codes supplemented by clinical documentation should accompany definitional standardisation.

Design of culturally adapted screening tools and intervention protocols assumes particular importance given the limited Asia-Pacific representation (21.9%) and the distinctive patterns observed in the Singapore studies. Research should establish if Western patterns generalise globally or require context-specific adaptation. There is a clear need for long-term outcome studies to better understand the trajectories of ARFAs and to assess whether observed intervention effects are sustained beyond the 12-month follow-up periods that dominate the current literature. Short-term reductions in ED utilisation provide an incomplete picture of recovery for this population. Future studies should therefore adopt broader outcome frameworks that extend beyond healthcare utilisation to include substance use recovery, social functioning, and quality of life (QoL). From a policy perspective, this shift is critical: interventions that reduce ED visits without improving functional or social outcomes may deliver limited long-term value and risk displacement.

Similarly, we should also emphasise economic evaluation. Robust cost-effectiveness analyses are essential to inform policy decisions in resource-constrained healthcare systems in Singapore. Such evaluations should capture not only the direct costs of intervention delivery and downstream healthcare savings but also wider societal impacts, including criminal justice involvement, emergency medical services (EMS) utilisation, employment and productivity changes, and housing stability. For Singapore, where health system financing and inter-agency coordination span healthcare, social services, and public safety sectors, this broader economic lens is particularly relevant.

Beyond effectiveness and cost, implementation science research is needed to bridge the gap between intervention efficacy and real-world scalability. Studies examining barriers and facilitators to adoption, for example, workforce capacity, training requirements, inter-agency coordination, data-sharing infrastructure, and organisational readiness, would provide critical guidance for health systems that seek to integrate ARFA interventions into routine care.

Most importantly, there is a pressing need for rigorous randomised controlled trials (RCTs) with adequate sample sizes, appropriate comparator conditions, and comprehensive outcome measurement. The current interventions are dominated by observational designs, which are vulnerable to bias from regression to the mean, selection effects, and unmeasured system-level influences. This limits confidence in causal inference and constrains the development of evidence-based policy. Future trials should therefore incorporate longer follow-up periods, assess both utilisation-based and patient-centred outcomes, and embed formal economic evaluations. Such trials would provide the level of evidence required to justify sustained investment, guide resource allocation, and support the integration of effective ARFA interventions into mainstream healthcare policy and service delivery.

## 5. Conclusions

This scoping review shows that progressing in the ARFA field requires coordinated efforts to develop validated definitions, extend research into non-Western contexts, and conduct rigorous RCTs of promising interventions. Without these advances, improvements in care delivery and resource utilisation for ARFAs will remain fragmented and difficult to sustain. Addressing these foundational gaps could help healthcare systems transition from costly reactive care toward proactive, effective interventions for this vulnerable population.

## Figures and Tables

**Table 1:** Distribution of study designs by ARFA classification (n = 73).

Study Design	Pure ARFA (%)	FA + ARFA (%)	Total (%)
Retrospective Cohort Study	6 (8.2)	36 (49.3)	42 (57.5)
Cross-Sectional Study	0 (0.0)	11 (15.1)	11 (15.1)
Non-Randomised Controlled Trial	2 (2.7)	6 (8.2)	8 (11.0)
Qualitative Study	5 (6.8)	1 (1.4)	6 (8.2)
Mixed Methods	0 (0.0)	3 (4.1)	3 (4.1)
Case-Control Study	0 (0.0)	2 (2.7)	2 (2.7)
Prospective Cohort Study	1 (1.4)	0 (0.0)	1 (1.4)
**Total**	**14 (19.2)**	**59 (80.8)**	**73 (100.0)**

ARFA = alcohol-related frequent attenders; FA + ARFA = frequent attenders with alcohol subgroup. Non-randomised controlled trial includes pre–post treatment effects without randomisation, case management, assertive community treatment, and integrated care programmes.

**Table 2:** Distribution of frequency thresholds by ARFA classification (n = 73).

Threshold Definition	Pure ARFA (%)	FA + ARFA (%)	Total (%)
≥2 visits/year	2 (2.7)	1 (1.4)	3 (4.1)
≥3 visits/year	0 (0.0)	10 (13.7)	10 (13.7)
≥4 visits/year	1 (1.4)	15 (20.5)	16 (21.9)
≥5 visits/year	2 (2.7)	10 (13.7)	12 (16.4)
≥6 visits/year	1 (1.4)	2 (2.7)	3 (4.1)
≥10 visits/year	4 (5.5)	2 (2.7)	6 (8.2)
Other/Top percentile	4 (5.5)	19 (26.0)	23 (31.5)
**Total**	**14 (19.2)**	**59 (80.8)**	**73 (100.0)**

ARFA: alcohol-related frequent attenders; FA + ARFA: frequent attenders with alcohol subgroup. Other/Top percentile: percentile-based selection (top 20 most frequent users), combined time-period criteria (≥10 visits/year or ≥5 visits/3 months), tiered frequency categories (repeat users 4–7 visits, highly frequent 8–18 visits, super frequent ≥ 19 visits), variable thresholds across study duration, non-standard timeframes (visits per 2 years or per 72 h period), very high thresholds (12–20 visits/year), and studies without specific numeric cut-offs.

**Table 4:** Characteristics and outcomes of intervention studies for ARFAs (n = 8).

Author (Year)	Sample Size	Study Design	Country	Follow-Up Period	Key Outcomes
Chiang (2014) [21]	14	Pre–post	Taiwan	6 months	Mean ED visits decreased from 10.5 to 4.36 visits/patient/month (58.3% reduction, *p* = 0.004); pain management group: 14.9 to 5.79 visits/month (*p* = 0.031); chronic disease group: 6.1 to 2.9 visits/month (*p* < 0.001)
Hedayioglu (2020) [22]	24	Pre–post; mixed methods	UK	12 months	Demonstrated value of case management approach; outcomes measured included quality of life, anxiety, and loneliness; specific quantitative results not fully reported
Lintzeris (2020) [23]	46	Pre–post	Australia	6 months	Significant reductions in preventable ED presentations (*p* < 0.05), non-preventable ED presentations (*p* < 0.01), hospital admissions (*p* < 0.01), and associated costs; significant reduction in primary substance use days (*p* < 0.01)
Scheiner (2019) [24]	101	Pre–post	UK	12 months	Highly significant mean reduction in ED attendances (*p* < 0.0001); lower gains in patients with alcohol-related presentations compared to other FAs
Sathyanarayanan (2021) [25]	29	Pre–post	US	12 months	Total ED visits reduced from 181 to 110 (*p* < 0.001); ED visits per patient/month: 0.52 to 0.31 (*p* < 0.001); inpatient visits per patient/month: 0.10 to 0.03 (*p* < 0.01)
Elston (2022) [26]	808	Pre–post	UK	12 months	ED attendance IRR = 0.856 at 12 months; ED admission IRR = 0.871; moderate effectiveness in reducing attendances and length of stay
Wu et al. (2024) [27]	93	Protocol/Pre–post (Ongoing)	Singapore	12 months	Protocol paper reporting baseline characteristics; 93 ARFAs recruited from 3 hospitals; predominantly male (93.6%); median age 57 y; 66.7% unemployed; 52.7% with liver disease; outcomes pending trial completion
Mak et al. (2022) [28]	14	Pre–post	Singapore	6 months	45.3% reduction in ED visits post-intervention (*p* = 0.025); demonstrated feasibility of ACT model for ARFA population in Singapore context

ACT = Assertive Community Treatment; IRR = Incidence Rate Ratio.

## Data Availability

No new data were created or analyzed in this study.

## Supplementary Materials

The following supporting information can be downloaded at: https://www.mdpi.com/article/10.3390/jcm15134892/s1, Table S1: Characteristics of the 73 studies included in the scoping review.
