# Supplementary material for: Alcohol-Related Frequent Attenders to Emergency Departments: A Scoping Review with Implications for Singapore
Source: J Clin Med. 2026 Jun 23;15(13):4892. doi: 10.3390/jcm15134892 (PMC13362420; doi:10.3390/jcm15134892)
Supplement: Supplementary file 1 [file jcm-15-04892-s001.zip › jcm-4364985-supplementary.pdf]

Supplementary Table S1. Characteristics of the 73 studies included in the scoping review.

No.	Study	Title	Country	Study Design	Sample Size	Frequency Threshold / Definition
1	Aagaard 2014	Predictors of frequent visits to a psychiatric emergency room: A large-scale register study combined with a small-scale interview study	Denmark	Mixed Methods	8,034	≥ 5 visits / year
2	Aguglia 2023	Sociodemographic and Clinical Correlates Associated with the Frequent Service Users in an Italian Psychiatric Emergency Department	Italy	Observational - Cohort Study; Quantitative Study	549	≥ 3 visits / year
3	Al-Surimi 2021	Epidemiology of frequent visits to the emergency department at a tertiary care hospital in Saudi Arabia: Rate, visitors' characteristics, and associated factors	Saudi Arabia	Observational - Cohort Study	82,046	≥ 4 visits / year
4	Andren 1985	Heavy users of an emergency department: psycho-social and medical characteristics, other health care contacts and the effect of a hospital social worker intervention	Sweden	Observational - Cohort Study; Quantitative Study	232	≥ 4 vistis / year
5	Capp 2013	Characteristics of Medicaid enrollees with frequent ED use	United States	Other: Retrospective chart review	1,619	≥ 4 vistis / year
6	Castner 2015	Frequent Emergency Department Utilization and Behavioral Health Diagnoses	United States	Observational - Cohort Study	56,491	≥ 3 visits / year
7	Cawley 2021	Understanding the 100 highest users of health and social services in San Francisco	United States of America	Observational - Cohort Study	100	≥ 4 visits / year
8	Chiang 2014	Dynamic internet-mediated team-based case management of highfrequency emergency department users	Taiwan	Interventional - Non Randomised Control Trial	14	Other: three or more times within a 72-hour period in a month
9	Chiu 2020	Persistent frequent emergency department users with chronic conditions: A population-based cohort study	Quebec, Canadaf	Observational - Cohort Study; Quantitative Study	297,182	≥ 4 vistis / year
10	Chiu 2022	Profiles of frequent emergency department users with chronic conditions: a latent class analysis	Canada	Observational - Cohort Study	451,775	≥ 5 visits / year
11	Curran 2008	The association of psychiatric comorbidity and use of the emergency department among persons with substance use disorders: an observational cohort study	USA	Observational - Cohort Study	7,570	Other: > 4+, 8+, 12+, 16+, or 20+ visits over the span of the study (4 year study)
12	Dent 2010	The impact of frequent attenders on a UK emergency department	UK	Observational - Cohort Study	44	>= 10 visits / year
13	Dinh 2016	Trends and characteristics of short-term and frequent representations to emergency departments: A population-based study from New South Wales, Australia	Australia	Observational - Cross Sectional	4,188,283	
14	DiPietro 2012	Ill, itinerant, and insured: the top 20 users of emergency departments in Baltimore city	USA	Observational - Cohort Study	20	
15	Dufour 2020	Frequent emergency department use by older adults with ambulatory care sensitive conditions: A population-based cohort study	Canada	Observational - Cohort Study	264,473	≥ 4 vistis / year

No.	Study	Title	Country	Study Design	Sample Size	Frequency Threshold / Definition
16	Elston 2022	Effectiveness of a targeted telephone-based case management service on activity in an Emergency Department in the UK: a pragmatic difference-in-differences evaluation	United Kingdom	Interventional - Non-Randomised Control Trial	808	
17	Fleury 2022	Predictors of Frequent Emergency Department Use and Hospitalization among Patients with Substance-Related Disorders Recruited in Addiction Treatment Centers	Canada	Observational - Cohort Study	17,819	≥ 3 visits / year
18	Fleury 2022	Predictors of no, low and frequent emergency department use for any medical reason among patients with cannabis-related disorders attending Quebec (Canada) addiction treatment centres	Canada	Observational - Cohort Study	9,836	≥ 3 visits / year
19	Fleury 2023	Predictors of dropout from treatment among patients using specialized addiction treatment centers	Canada	Observational - Cohort Study	16,179	≥ 3 visits / year
20	Garde 2024	Functional somatic symptoms in Emergency Department frequent presenters	Australia	Observational - Cohort Study	638	
21	Giannouchos 2019	Predictors of Multiple Emergency Department Utilization Among Frequent Emergency Department Users in 3 States	United States	Observational - Cohort Study	1,003,626	≥ 5 visits / year
22	Giannouchos 2025	Is frequent emergency department use a complement or substitute for other healthcare services? Evidence from South Carolina Medicaid enrollees	USA	Observational - Cohort Study	52,845	
23	Goh 2022	Why Are Some Male Alcohol Misuse Disorder Patients High Utilisers of Emergency Health Services? An Asian Qualitative Study	Singapore	Qualitative Study	20	≥ 5 visits / year
24	Hall 2015	EMS-STARS: Emergency Medical Services 'Superuser' Transport Associations: An Adult Retrospective Study	USA	Observational - Cross Sectional	427	
25	Hamilton 2014	Imaging of frequent emergency department users with alcohol use disorders	United States	Observational - Cohort Study	51	≥ 10 visits / year
26	Hansagi 2012	Predictors of repeated emergency department visits among persons treated for addiction	Sweden	Observational - Cohort Study	1,287	≥ 2 visits / year
27	Harring 2024	Frequent contacts to Emergency Medical Services (EMS): more than frequent callers	Norway	Observational - Cross Sectional	2,149,400	
28	Hedayioglu 2020	Managing the needs of frequent attenders of urgent care services: a case management approach	England	Interventional - Non Randomised Control Trial; Mixed Methods	24	Other: Not specifically defined, but recruited sample minimum 5 visits.
29	Hulme 2020	Mortality among patients with frequent emergency department use for alcohol-related reasons in Ontario: a population-based cohort study	Canada	Observational - Cohort Study	25,813	≥ 2 visits / year
30	Jacob 2016	Designing services for frequent attenders to the emergency department: a characterisation of this population to inform service design	United Kingdom	Observational - Cohort Study; Observational - Case Report	2,463	≥ 5 visits / year

No.	Study	Title	Country	Study Design	Sample Size	Frequency Threshold / Definition
31	Kelekar 2025	Age-Based Variations in Frequent Emergency Department (ED) Use Among Older Adults: An Analysis Using the New York University-Johns Hopkins University Emergency Department Algorithm (NYU/JHU-EDA)	USA	Observational - Cross Sectional; Quantitative Study	417,240	≥ 4 visits / year
32	Kim 2024	Characteristics of consecutive versus nonconsecutive frequent emergency medical services transport to a single emergency department	South Korea	Observational - Cohort Study	patients: 205 number of frequent EMS transport: 1,204	≥ 3 visits / year
33	Klein 2018	Emergency Department Frequent Users for Acute Alcohol Intoxication	United States	Observational - Cohort Study; Other: Retrospective cohort study	325	>=20 visits / year
34	Kuek 2019	Characteristics of Frequent Users of Emergency Medical Services in Singapore	Singapore	Observational - Cohort Study (retrospective)	243	≥ 4 EMS conveyances / year
35	Kyle 2021	The wider implications of the COVID-19 pandemic: Assessing the impact of accident and emergency use for frequent attenders	United Kingdom	Observational - Cohort Study	2019 Cohort (n=80) 2020 Cohort (n=80)	
36	Laine 2005	Availability of medical care services in drug treatment clinics associated with lower repeated emergency department use	New York	Observational - Cohort Study	8,397	≥ 2 visits / year
37	Lee 2020	Characteristics of frequent adult emergency department users: A Korean tertiary hospital observational study	South Korea	Observational - Cross Sectional	29,759	≥ 4 vistis / year
38	Lee 2022	Characteristics and Resource Utilization Associated with Frequent Users of Emergency Departments	Taiwan	Observational - Cohort Study	2,191 Frequent Users	≥ 4 visits / year
39	Lennard 2022	Seizures and emergency department: characteristics and factors of repeat adult attendees	England (UK)	Observational - Cohort Study	450	
40	Leporatti 2016	Targeting frequent users of emergency departments: Prominent risk factors and policy implications	Italy	Observational - Cohort Study	14,7864	≥ 3 visits / year
41	Lintzeris 2020	Evaluation of an Assertive Management and Integrated Care Service for Frequent Emergency Department Attenders with Substance Use Disorders: The Impact Project: Evaluating an assertive management service for frequent ED attenders with substance use disorders	Australia	Interventional - Non Randomised Control Trial	46 (34 clients, 12 in the comparison group)	≥ 5 visits / year
42	Liu 2013	Frequent ED users: are most visits for mental health, alcohol, and drug-related complaints?	United States	Observational - Cohort Study	2,496	Other: repeat users (4-7visits) highly frequent users (8-18 visits) super frequent users(≥19 visits)
43	Locker 2007	Defining frequent use of an urban emergency department	United Kingdom	Observational - Cohort Study	75,141	
44	Mak 2022	A new paradigm in management of frequent attenders to emergency departments with severe alcohol use disorder—A pilot study for assertive community treatment in Singapore	Singapore	Interventional - Non Randomised (prospective pre-post)	14	≥ 6 alcohol-related ED visits / year
45	Mandelberg 2000	Epidemiologic analysis of an urban, public emergency department's frequent users	United States	Observational - Cross Sectional; Observational - Cohort Study	43,383	≥ 5 visits / year

No.	Study	Title	Country	Study Design	Sample Size	Frequency Threshold / Definition
46	McCormack 2015	Including frequent emergency department users with severe alcohol use disorders in research: assessing capacity	New York	Other: Feasibility study on prospective ED patients	20	≥ 4 vistis / year
47	McCormack 2015	Voices of homeless alcoholics who frequent bellevue hospital: a qualitative study	New York City, USA	Qualitative Study	20	≥ 4 vistis / 2 year
48	Meng 2017	Disordered lives: Life circumstances and clinical characteristics of very frequent users of emergency departments for primary mental health complaints	Canada	Qualitative Study; Mixed Methods; Other: Retrospective thematic analysis	34	≥ 10 visits / year
49	Minassian 2013	Frequent emergency department visits are more prevalent in psychiatric, alcohol abuse, and dual diagnosis conditions than in chronic viral illnesses such as hepatitis and human immunodeficiency virus	United States	Observational - Cohort Study; Quantitative Study	39,249	≥ 4 vistis / year
50	Moe 2021	Characteristics of frequent emergency department users in British Columbia, Canada: a retrospective analysis	Canada	Observational - Cohort Study	1,196,353	Other: no cut off
51	Moe 2021	Identifying subgroups and risk among frequent emergency department users in British Columbia	British Columbia (Canada)	Observational - Cohort Study; Quantitative Study	58,491	≥ 3 visits / year
52	Moe 2022	Subgroups of people who make frequent emergency department visits in Ontario and Alberta: a retrospective cohort study	Ontario and Alberta (Canada)	Observational - Cohort Study	ranged from 435,334 to 477647 each year in Ontario, and from 98,840 to 105047 in Alberta	≥ 4 visits / year
53	Monti 2022	Data mining-based clinical profiles of substance use-related emergency department utilizers	USA	Observational - Cross Sectional	199	≥ 3 visits / year
54	Neale 2017	Socio-demographic characteristics and stereotyping of people who frequently attend accident and emergency departments for alcohol-related reasons: Qualitative study	London, United Kingdom	Qualitative Study	30	Other: Potential participants were defined as all patients who had attended A&E 10 times within the last year or 5 times in the last three months for an alcohol-related condition
55	Ng 2023	Characteristics of individuals who frequently use emergency departments in Hong Kong: a regionbased cohort study	Hong Kong	Observational - Cohort Study	215,862	
56	Oliveras 2024	Another Round: Influence of Alcohol-Related Conditions and Other Drug Use-Related Disorders in Emergency Department Frequent Use - A Single-Site Matched Case-Control Study in Spain	Spain	Other	1,218	≥ 5 visits / year
57	Oliveras 2024	Effects of alcohol-related problems on the costs of frequent emergency department use: an economic analysis of a case-control study in Spain	Spain	Other	1,218	≥ 5 visits / year
58	Parkman 2017	How Do People Who Frequently Attend Emergency Departments for Alcohol-Related Reasons Use, View, and Experience Specialist Addiction Services?	London, United Kingdom	Qualitative Study	30	Other: ≥10x per year or ≥5x in 3 months

No.	Study	Title	Country	Study Design	Sample Size	Frequency Threshold / Definition
59	Parkman 2017	Qualitative exploration of why people repeatedly attend emergency departments for alcohol-related reasons	London, United kingdom	Qualitative Study	30	Other: Inclusion criteria for the study were "any patient aged 16 or over who attends any Accident and Emergency de- partment 10 or more times within a year or 5 or more times within a 3-month period for an alcohol-related condition"
60	Phillips 2006	The effect of multidisciplinary case management on selected outcomes for frequent attenders at an emergency department	Australia	Observational - Cohort Study; Other: retrospective	60	>= 6 visits / year
61	Purdie 1981	The chronic emergency department patient	United States	Observational - Cohort Study	16	12 visits/year
62	Quilty 2016	Factors contributing to frequent attendance to the emergency department of a remote Northern Territory hospital	Australia	Observational - Cross Sectional	227	≥ 6 visits / year
63	Ramasubbu 2016	Profile of Frequent Attenders to a Dublin Inner City Emergency Department	Dublin, Ireland	Observational - Cohort Study	20	Other: top 20 most frequent presenters to the ED
64	Samosh 2024	Community Mental Health Services for Frequent Emergency Department Users: A Qualitative Study of Outcomes Perceived by Program Clients and Case Managers	Canada	Qualitative Study	15 program clients, 6 case managers	
65	Sathyanarayanan 2021	Reducing Frequency of Emergency Department and Inpatient Visits Through Focused Case Management	United States	Interventional - Non-Randomised Control Trial	29	≥ 3 visits / year
66	Scheiner 2019	The effect of integrated care on self-management and emergency department attendance	UK	Interventional - Non Randomised Control Trial	40 and 61	15 visits/year
67	Shankar 2022	High touch, high trust: Using community health advocates and lawyers to address ED high utilizers	USA	Observational - Cohort Study	151	≥ 4 visits / year
68	Urbanoski 2018	Frequent use of emergency departments for mental and substance use disorders	Canada	Observational - Cross Sectional	5,416	≥ 5 visits / year
69	Vu 2015	Screening of mental health and substance users in frequent users of a general Swiss emergency department	Switzerland	Observational - Cross Sectional	399	≥ 4 vistis / year
70	Wise-Harris 2017	"Hospital was the Only Option": Experiences of Frequent Emergency Department Users in Mental Health	Canada	Mixed Methods	166	≥ 5 visits / year
71	Woo 2016	Frequent Users of Hospital Emergency Departments in Korea Characterized by Claims Data from the National Health Insurance: A Cross Sectional Study	South Korea	Observational - Cross Sectional	156,246	≥ 4 vistis / year
72	Wu 2024	Assertive community treatment for high-utilizing alcohol misuse patients: a before-and-after cohort study protocol	Singapore	Interventional - Non-Randomised Control Trial	Target recruitment ~34 patients annually, total ongoing recruitment reported as 93 as of March 2023 in 3 sites	≥ 5 visits / year

No.	Study	Title	Country	Study Design	Sample Size	Frequency Threshold / Definition
73	Zhang 2022	Emergency department presentations of patients with alcohol use disorders in an Australian regional health district	Australia	Observational - Cohort Study	2,519	

ARFA = alcohol-related frequent attenders; FA+ARFA = frequent attender studies reporting alcohol-specific subgroups or outcomes; ED = emergency department; EMS = emergency medical services.
